# The External Electric Field-Induced Tunability of the Schottky Barrier Height in Graphene/AlN Interface: A Study by First-Principles

**DOI:** 10.3390/nano10091794

**Published:** 2020-09-09

**Authors:** Xuefei Liu, Zhaocai Zhang, Bing Lv, Zhao Ding, Zijiang Luo

**Affiliations:** 1 Key Laboratory of Low Dimensional Condensed Matter Physics of Higher Educational Institution of Guizhou Province, Guizhou Normal University, Guiyang 550025, China; 201307129@gznu.edu.cn (X.L.); lvbing@gznu.edu.cn (B.L.); 2 Beijing Institute of Space Science and Technology Information, Beijing 100094, China; zhangzhaocai@gmail.com; 3 College of Big data and Information Engineering, Guizhou University, Guiyang 550025, China; zding@gzu.edu.cn; 4 College of Information, Guizhou University Finance and Economics, Guiyang 550025, China

**Keywords:** graphene/AlN interface, vertical strain, Schottky barrier height

## Abstract

Graphene-based van der Waals (vdW) heterojunction plays an important role in next-generation optoelectronics, nanoelectronics, and spintronics devices. The tunability of the Schottky barrier height (SBH) is beneficial for improving device performance, especially for the contact resistance. Herein, we investigated the electronic structure and interfacial characteristics of the graphene/AlN interface based on density functional theory. The results show that the intrinsic electronic properties of graphene changed slightly after contact. In contrast, the valence band maximum of AlN changed significantly due to the hybridization of Cp and Np orbital electrons. The Bader charge analysis showed that the electrons would transfer from AlN to graphene, implying that graphene would induce acceptor states. Additionally, the Schottky contact nature can be effectively tuned by the external electric field, and it will be tuned from the p-type into n-type once the electric field is larger than about 0.5 V/Å. Furthermore, the optical absorption of graphene/AlN is enhanced after contact. Our findings imply that the SBH is controllable, which is highly desirable in nano-electronic devices.

## 1. Introduction

Due to their fascinating optoelectronic properties, two-dimensional (2D) materials such as graphene [1,2], transition metal dichalcogenides [3,4,5,6], phosphorene [7,8], carbon nitride [9,10,11], and III-Nitrides (III-N) [12,13,14,15,16] have attracted extensive attention during the past years. Among these 2D materials, graphene, as a pioneering representative, shows room-temperature mobilities of ~10,000 cm^2^/V·s [1], which is rather desirable for next generation nanodevices.

However, shown without a gap, graphene itself is rather constrained in practical applications. Fortunately, the construction of the heterojunction by vertically stacking two 2D materials provides a new avenue to extend the application possibility of their individual components [17,18,19]. The underlying physics is due to the lack of dangling bonds at the interface as well as the weak interactions of two sublayers [20]. In recent years, more and more work on graphene-based van der Waals (vdW) heterostructures have been reported [21,22,23,24,25,26]. These reports prove that the intrinsic optoelectronic properties of the individual components are preserved after contacting them. More than that, some newly novel properties have also been found in the vertical stacking heterojunctions.

AlN is a rather significant wide bandgap semiconductor due to its small or even negative electron affinity, excellent chemical stability, a superior mechanical strength, and a high thermal conductivity [27,28]. Furthermore, AlN has also been confirmed as a potential optoelectronic material in the deep ultraviolet light region [29]. These excellent properties of the bulk phase AlN have naturally inspired people to explore its various properties in the 2D phase. Recently, 2D AlN few-layer sandwiched between the graphene and Si substrates as well as an ultrathin aluminum nitride (AlN) nanosheet were successfully fabricated experimentally [16,30]. As is well known, an excellent electrode to a 2D material-based field-effect transistor (FET) is rather important. Usually, a conventional metal-semiconductor contact would not only induce a Schottky barrier height (SBH), but also induce some interfacial states, both of which would degrade the interface performance [31]. Graphene with metallicity is an ideal electrode for 2D materials due to its lack of dangling bonds and weak interaction with the semiconductor. Thus, investigating the graphene/AlN heterojunction is interesting, especially for the graphene/AlN-based nano-FET. Recently, Sciuto et al. have studied the graphene/bulk-AlN rather than 2D AlN heterojunction properties, and predicted that the polarity and surface reconstruction of nitride could effectively tune the Fermi-level [32]. In addition, we previously studied how biaxial strain as well as the defects would tune the electronic properties of the graphene/AlN heterojunction by first-principles calculations [21]. However, for the vdW heterojunction, modulating the SBH is an effective method to reduce the interfacial resistance. The external electric field has been proven to be an important approach to tune its SBH [33,34]. Theoretically, people can control the value and direction of the electric field to the heterojunction by setting a proper parameter in the input file of VASP. In the experiment, when nano-FET based on graphene/AlN is fabricated, graphene is the electrode, and the electric field can be applied across the interface by an external voltage. Nevertheless, how the external field would tune the interface properties of the graphene/AlN interface is still unclear. Thus, motivated by this, in this paper, we further investigated the graphene/AlN heterojunction properties by applying an external electric field based on the first-principles calculations. We found that the electric field would effectively modulate the Schottky barrier height (SBH) of the graphene/AlN heterojunction, which is desired to degrade the contact resistance between the grapheme (electrode) and AlN (tunnel). Our findings will provide valuable guidance for researchers to fabricate graphene/AlN-based devices.

## 2. Computation Method

The Vienna ab-initio simulation package (VASP) [35], which has implemented the generalized gradient approximation (GGA) [36] method was used to carry out the calculations. The Perdew–Burke–Ernzerhof (PBE) functional was utilized as the exchange-correlation potential and the projector augmented wave [37] (PAW) method was employed to describe the ion-electron interactions. The self-consistency total energy difference was set to$\;10^{-5}$ eV, the plane-wave energy cutoff was set to 500 eV, and the maximum Hellmann–Feynman force on each atom was less than 0.01 eV/Å, respectively. To correct the van der Waals interaction between the AlN and graphene sublayers, the DFT-D3 approach proposed by Grimme [38] was employed. A 20 Å vacuum thickness was constructed to cancel the interactions from the periodic images along the z-direction [39,40]. The Γ-centered Monkhorst–Pack [41] approach was used to sample the reciprocal space, and the grid density was set as 4 × 4 × 1. In order to cancel the errors of electrostatic potential, the total energy, and the atomic force under the periodic boundary condition, the dipole correction was considered in this work [42]. The band structure analysis was conducted by using VASPKIT [43]. The heterostructure was constructed automatically with our python code *heterojunction*. To describe the stability of the heterostructure, the binding energy is defined by Equation (1) [18,44]:

(1) $$E_{b}=\left(E_{graphene/AlN}-E_{graphene}-E_{AlN}\right)/A$$

where $E_{b}$ is the heterojunction binding energy; $E_{graphene/AlN}$ represents the total energy of the heterostructure$;\;E_{graphene}$ and $E_{AlN}$ are the total energy of graphene and AlN monolayer, separately. *A* denotes the x–y in-plane area of the heterojunction.

## 3. Results and Discussion

### 3.1. Structural Properties

**Figure 1:**
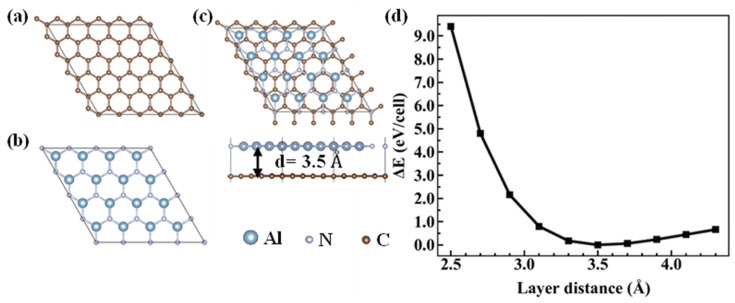
(**a**) The 5×5 supercell of graphene, (**b**) the 4×4 supercell of AlN, (**c**) the top view (upper) and side view (lower) of graphene/AlN heterojunction. (**d**) The energy difference with respect to the most stable structure of the graphene/AlN system as a function of interlayer distance.

We first optimized the graphene and AlN primitive cells, and the lattice constants of graphene and AlN were $a_{1}$ = $b_{1}$ = 2.46 Å, $a_{2}$ = $b_{2}$ = 3.08 Å, respectively, agreeing well with that in [33,45]. By using our python code *heterojunction,* the graphene/AlN heterojunction was built with a new lattice constant of *a* = *b* = 12.33 Å and the lattice mismatch was lower than 1%. In other words, the heterostructure was constructed by a 5 × 5 of graphene and a 4 × 4 AlN supercell, as depicted in Figure 1. As the total energy of the heterojunction is relevant to the interlayer distance, by changing the interlayer distance from 2.5 to 4.3 Å, we found that the favorably energetic layer distance was 3.5 Å, and the binding energy of the graphene/AlN interface based on Equation (1) was −14.1 meV/${Å}^{2}$, indicating that graphene/AlN is a typical vdW heterostructure [18,44].

### 3.2. Electronic Properties

**Figure 2:**
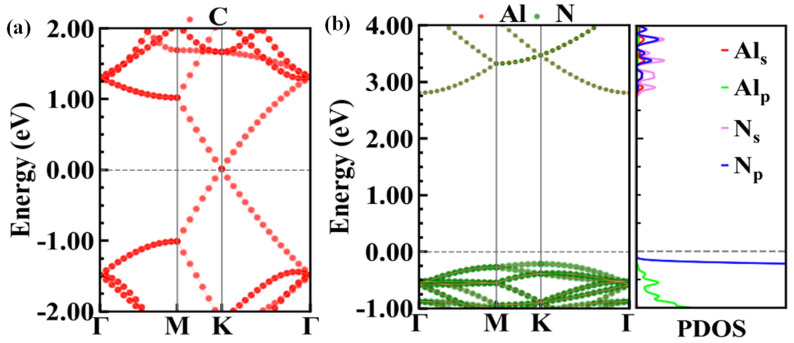
(**a**) The projected band structure of graphene, (**b**) the projected band structure and density states of 2D AlN.

We first calculated the electronic structures of the graphene and monolayer AlN, as shown in Figure 2.

The typical Dirac-point of graphene is found to occur at the Fermi level. The conduction band minimum (CBM) of AlN rides at the Γ point while the valence band maximum (VBM) is found at the K point, implying an indirect bandgap of 3.1 eV, which agreed well with our previous PBE calculations [21]. The projected density of states (PDOS) indicates the VBM is mainly contributed by N_p_ orbital electrons while the CBM is contributed by N_s_ orbital electrons.

**Figure 3:**
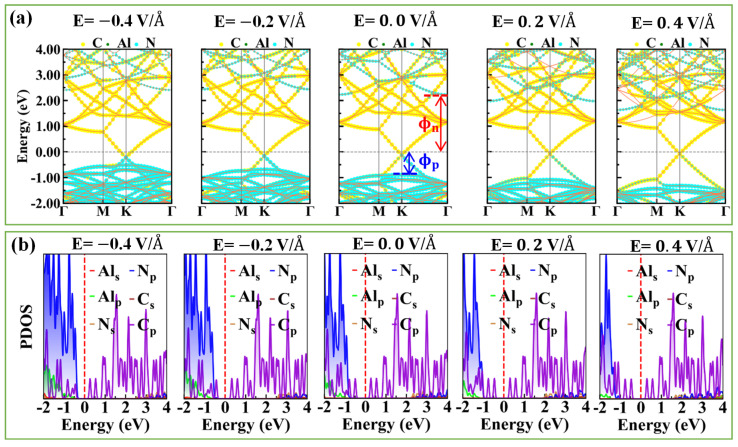
(**a**) The projected band structure of the graphene/AlN heterojunction under external electric field ranging from −0.4 V/Å to 0.4 V/Å. (**b**) The projected density of states of graphene/AlN heterostructure under the corresponding external electric field. Fermi level was aligned to 0 eV. The Schottky barrier height (SBH) is defined in Figure 3a, where $\Phi_{n}$ is the n-type SBH, and $\Phi_{p}$ is the p-type SBH.

**Figure 4:**
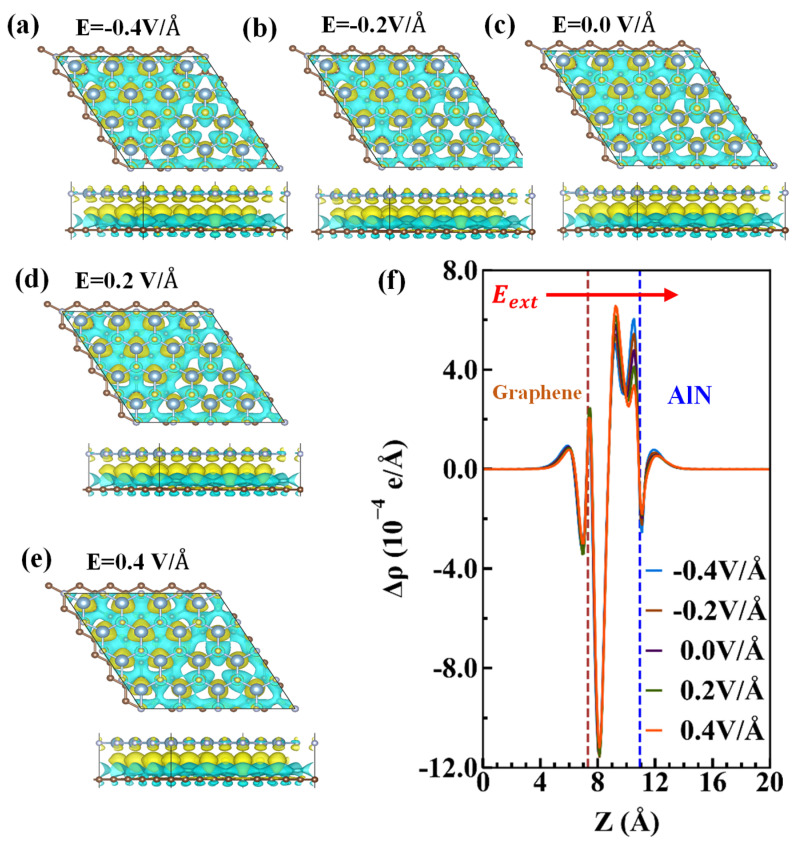
(**a**–**e**) The difference charge density of the graphene/AlN system under a different external electric field. The iso-surface was set to $3\times 10^{-4}$ e/${Å}^{3}$. The yellow and cyan colors represent the depletion and accumulation of electrons, respectively. (**f**) The averaged difference in the electron density for the graphene/AlN heterojunction under various external electric fields, the location of graphene, and the AlN sublayer are marked as brown and blue dashed lines, respectively. The positive external field direction is denoted by a red arrow.

**Figure 5:**
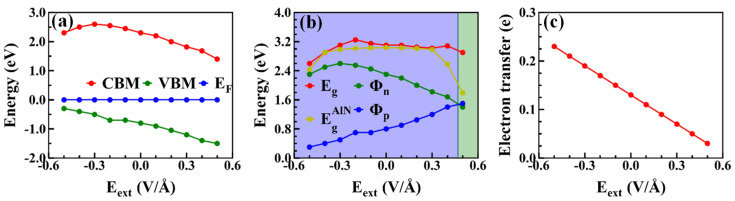
(**a**) The varying trends of CBM, VBM, and Fermi level E_F_ with respect to the external electric field. (**b**) The Schottky barriers $\Phi_{n}$, $\Phi_{p}$, and bandgap $E_{g}$ ($E_{g}^{AlN})\;$ as a function of the external electric field. (**c**) The electron transfer amount from AlN to graphene as a function of the external field.

The work function is defined as $WF=E_{vac}-E_{F}$, where $E_{vac}$ and $E_{F}$ are the vacuum energy and Fermi energy, respectively. Based on the definition, the work function for graphene and AlN are 4.30 and 5.11 eV, respectively. Applying the external electric field is an approach to modulate the electronic properties of the vdW interface [46]. The projected band structures and density of states (PDOS) of the heterojunction under different external electric fields were investigated, as shown in Figure 3. Herein, we applied an external electric field in the direction perpendicular to the x–y plane of the graphene/AlN heterojunction and defined the positive direction was from graphene to AlN, as shown in Figure 4f. Compared to Figure 2, it was found that the intrinsic band structure of graphene was almost preserved due to the weak interaction in the vdW heterojunction, but the VBM of AlN changed due to the charge redistribution between in the interface, as confirmed by the difference charge density illustrated in Figure 4. Concerning the PDOS in Figure 3b, we found that the underlying physics regarding the charge redistribution resulted from the strong hybridization of the Np and Cp orbital electrons. In contrast, with negligible hybridization around the CBM energy region, the CBM of AlN was almost unchanged. As shown in Figure 3, with the increase in the negative electric field, more and more electrons were transferred from AlN to graphene. This was confirmed by the downward shift of the Dirac-point with respect to the Fermi level. These results prove that graphene induced acceptor states, as confirmed by the charge density difference and the Bader charge analysis [47] discussed in the following section. Consequently, the bandgap of the AlN sublayer in the heterostructure is changed dependently of the external electric field, as shown in Figure 5b.

The plane-averaged charge density difference (PCDD) between the graphene/AlN interface is represented by Equation (2) [19,21]:

(2) $$\Delta \rho =\rho_{graphene/AlN}-\rho_{graphene}-\rho_{AlN}\;$$

where $\rho_{graphene/AlN}$, $\rho_{AlN}$ and $\rho_{graphene}$ are the plane-averaged charge density of the graphene/AlN interface, AlN monolayer, and graphene monolayer, respectively. As depicted in Figure 4a, the PCDD results show the interaction and electron transfer between the AlN and graphene sublayers. The results show that charges were redistributed after contact between the graphene and AlN, that is, both charge accumulation and depletion mainly occurred in the middle of the interface, as confirmed by the x–y plane-averaged charge density difference curves along the z-direction (shown in Figure 4f), in which the positive and negative values represent the charge accumulation and depletion, respectively. Thus, graphene was found to obtain electrons from AlN, which would result in the formation of an interface dipole layer and its associated potential step [48,49]. We further investigated the charge transfer by using Bader’s method, and the results indicate that the charge transfers from AlN to graphene under an external electric field ranging from −0.5 V/Å to +0.5 V/Å are decreased from 0.23 e to 0.03 e linearly, as shown in Figure 5c, further indicating that graphene would induce acceptor states, which is consistent with the band structures.

### 3.3. Tunability of SBH under the External Electric Field

The Schottky barrier height (SBH) plays a vital role in the metal/semiconductor interfacial system or Schottky-based devices. In the theory of the Schottky–Mott model [50,51], the n-type and p-type Schottky barrier are represented by

(3) $$\Phi_{n}=\mathrm{CBM}-E_{F\;}$$

(4) $$\Phi_{p}=E_{F}-\mathrm{VBM}\;$$

separately, where $\Phi_{n}$ is the n-type SBH; $\Phi_{p}$ is the p-type SBH; and $E_{F}$ is the Fermi level, which is referred to as zero during the study. Without an external electric field, the $\Phi_{n}$ and $\Phi_{p}$ of graphene/AlN were found to be 2.3 eV and 0.8 eV, respectively. Hence, a p-type Schottky contact was built at the graphene/AlN interface.

Usually, a high SBH will seriously increase the contact resistance and further degrade the contact performance of the field-effect transistor (FET). Thus, the tunability of SBH is rather meaningful for Schottky devices. The common methods of modulating the SBH include in-plane strain engineering [52], vertical strain engineering [22,53] as well as the external electric field [24]. In order to identity how the SBH of the graphene/AlN interface can be tuned by the external electric field, in this study, we applied serials of electric field values ranging from −0.5 V/Å to +0.5 V/Å in the vertical direction. The negative sign indicates that the electric field is directed from AlN toward graphene. The CBM, VBM, and E_F_ shown in Figure 5a, as a function of an external electric field, were increased within the considered electric field range, but with different degrees. Consequently, as depicted in Figure 5b, the graphene/AlN was found to be a p-type Schottky contact before the external field was larger than about +0.5 V/Å, and after that, the interface turned from a p-type into an n-type Schottky contact. As seen in Figure 5b, the bandgaps of AlN would change dependently on the electric field, due to the different shift amount of the CBM and VBM under different electric field values. We also calculated the bandgap of AlN itself under different electric field values, marked by the yellow curves in Figure 5b. In addition, when the electric field in the negative direction was increased to −0.5 V/Å, the p-type SBH was only 0.3 eV, implying that the contact resistance was rather small. This indicates that graphene is an ideal electrode material for AlN. Our findings are helpful in understanding the operation mechanism of the graphene/AlN-based FET under a vertical electric field.

### 3.4. The Optical Properties

Finally, we discuss the optical absorption ability of the graphene/AlN heterojunction. It noteworthy that the traditional optical properties calculation approach for three-dimensional (3D) material needs to be corrected in the case of a two-dimensional (2D) material [54,55]. This originates from the fact that the dielectric function is dependent on the vacuum thickness [56]. Thus, in 2D materials, the optical conductivity $\sigma_{2D\left(\omega \right)}$ is an important parameter to describe the optical properties. Based on the Maxwell equation, the 3D optical conductivity is defined as [54]

(5) $$\sigma_{3D\left(\omega \right)}=i\left[1-\varepsilon \left(\omega \right)\right]\varepsilon_{0}\omega \;$$

where ε(ω) is the complex dielectric function described in [43]; $\varepsilon_{0}$ is the vacuum permittivity; and ω is the frequency of the incident wave. The in-plane 2D optical conductivity is then determined by

(6) $$\sigma_{2D\left(\omega \right)}=L\sigma_{3D\left(\omega \right)}$$

where *L* is the slab thickness in the graphene/AlN heterojunction. Finally, the normalized absorbance *A*(ω) is obtained

(7) $$A=\frac{Re_{\tilde{\sigma }\left(\omega \right)}}{{\left|1+\tilde{\sigma }\left(\omega \right)/2\right|}^{2}}\;$$

where $\tilde{\sigma }\left(\omega \right)=\sigma_{2D\left(\omega \right)}/\left(\varepsilon_{0}c\right)$ is the normalized conductivity; *c* is the speed of light; and *Re* stands for the real part.

**Figure 6:**
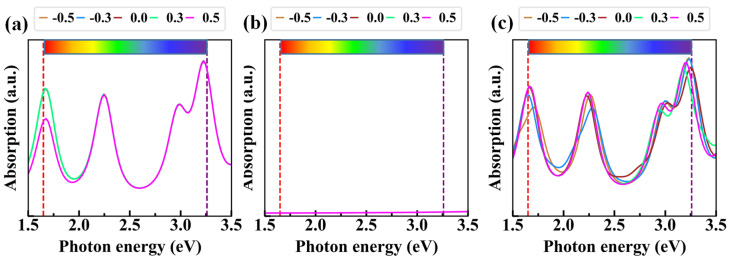
The absorption of (**a**) graphene, (**b**) AlN, and (**c**) the graphene/AlN heterojunction under a different external electric field.

Figure 6 illustrates the absorption as a function of the photon energy of the graphene/AlN heterojunction under various external electric fields. As is well known, the PBE method underestimated the bandgap but had almost no effect on band shape. Thus, we used our previous hybrid functional bandgap [57] (~4.04 eV) to analyze the optical properties. That is, based on the bandgap difference between PBE and the Heyd–Scuseria–Ernzerhof [58,59] (HSE) functional, the photon energy shifted with a scissor operator. The absorption was averaged in the x- and y-directions. The results show that the external electric field would affect the optical absorption accordingly, which is because the bandgap of AlN will change with the variation of the external electric field. With a rather larger HSE bandgap [57] (~4.04 eV), the monolayer AlN showed a rather weak absorption in the visible region, indicating that 2D AlN itself is not applicable for the optoelectronic field (i.e., photocatalysis) in the visible sunlight region. However, after being in contact with graphene, the absorption was significantly enhanced in the graphene/AlN heterojunction. Thus, we expect that this interface will be promising in nano-optoelectronic applications in the visible sunlight region.

## 4. Conclusions

In conclusion, the structural, electronic, and optical properties of the graphene/AlN interface under external electric field were investigated through density functional theory. The results of the projected band structure, the charge density differences, and the Bader charge showed that graphene acts as an acceptor while AlN acts as a donor. By applying the external electric field from −0.5 V/Å to +0.5 V/Å, we found that the electrons transferred from AlN to graphene would decrease. When the electric field was not applied across the interface, the graphene/AlN heterojunction was shown to be a p-type Schottky contact with a $\Phi_{p}$ of 0.8 eV. Additionally, we found that the Schottky barrier height could be tuned by the electric field effectively, that is, the $\Phi_{p}$ increased from 0.3 to 1.5 eV, while the $\Phi_{n}$ increased from 2.3 to 2.6 eV first, and after decreased to 1.4 eV. As a result, the graphene/AlN contact transforms from a p-type into an n-type with an electric field larger than about 0.5 V/Å. Furthermore, the optical calculations showed that the absorption will be enhanced in the visible region after contacting graphene and AlN. Our findings provide valuable guidance in the fabrication of graphene/AlN-based Schottky devices and promote the performance of nanodevices in the future.

## References

[1] Novoselov K.S., Geim A.K., Morozov S.V., Jiang D., Zhang Y., Dubonos S.V., Grigorieva I.V., Firsov A.A. (2004). Electric field effect in atomically thin carbon films. Science.

[2] Novoselov K.S., Geim A.K., Morozov S.V., Jiang D., Katsnelson M.I., Grigorieva I.V., Dubonos S.V., Firsov A.A. (2005). Two-dimensional gas of massless dirac fermions in graphene. Nature.

[3] Wang Q.H., Kalantar-Zadeh K., Kis A., Coleman J.N., Strano M.S. (2012). Electronics and optoelectronics of two-dimensional transition metal dichalcogenides. Nat. Nanotechnol..

[4] Duan X., Wang C., Pan A., Yu R., Duan X. (2015). Two-dimensional transition metal dichalcogenides as atomically thin semiconductors: Opportunities and challenges. Chem. Soc. Rev..

[5] Choi W., Choudhary N., Han G.H., Park J., Akinwande D., Lee Y.H. (2017). Recent development of two-dimensional transition metal dichalcogenides and their applications. Mater. Today.

[6] Qian Q., Zhang Z., Chen K.J. (2018). In situ resonant raman spectroscopy to monitor the surface functionalization of Mos2 and Wse2 for High-K integration: A first-principles study. Langmuir.

[7] Liu H., Neal A.T., Zhu Z., Luo Z., Xu X.F., Tomanek D., Ye P.D. (2014). Phosphorene: An unexplored 2D semiconductor with a high hole mobility. ACS Appl. Nano Mater..

[8] Carvalho A., Wang M., Zhu X., Rodin A.S., Su H., Castro Neto A.H. (2016). Phosphorene: From theory to applications. Nat. Rev. Mater..

[9] Naseri A., Samadi M., Pourjavadi A., Moshfegh A.Z., Ramakrishna S. (2017). Graphitic carbon nitride (G-C_3_n_4_)-based photocatalysts for solar hydrogen generation: Recent advances and future development directions. J. Mater. Chem. A.

[10] Ong W.-J., Tan L.-L., Ng Y.H., Yong S.-T., Chai S.-P. (2016). Graphitic carbon nitride (G-C_3_n_4_)-based photocatalysts for artificial photosynthesis and environmental remediation: Are we a step closer to achieving sustainability?. Chem. Rev..

[11] Niu H., Wang X., Shao C., Liu Y., Zhang Z., Guo Y. (2020). Revealing the oxygen reduction reaction activity origin of single atoms supported on G-C3n4 monolayers: A first-principles study. J. Mater. Chem. A.

[12] Singh A.K., Zhuang H.L., Hennig R.G. (2014). Ab initio synthesis of single-layer Iii-V materials. Phys. Rev. B.

[13] Zhang Z., Geng Z., Cai D., Pan T., Chen Y., Dong L., Zhou T. (2015). Structure, electronic and magnetic properties of hexagonal boron nitride sheets doped by 5D transition metal atoms: First-principles calculations and molecular orbital analysis. Physica E Low Dimens. Syst. Nanostruct..

[14] Kecik D., Onen A., Konuk M., Gürbüz E., Ersan F., Cahangirov S., Aktürk E., Durgun E., Ciraci S. (2018). Fundamentals, progress, and future directions of nitride-based semiconductors and their composites in two-dimensional limit: A first-principles perspective to recent synthesis. Appl. Phys. Rev..

[15] Al Balushi Z.Y., Wang K., Ghosh R.K., Vila R.A., Eichfeld S.M., Caldwell J.D., Qin X., Lin Y.C., DeSario P.A., Stone G. (2016). Two-dimensional gallium nitride realized via graphene encapsulation. Nat. Mater..

[16] Wang W., Zheng Y., Li X., Li Y., Zhao H., Huang L., Yang Z., Zhang X., Li G. (2019). 2D AlN layers sandwiched between graphene and si substrates. Adv. Mater..

[17] Zhang Z., Qian Q., Li B., Chen K.J. (2018). Interface engineering of monolayer Mos2/Gan Hybrid heterostructure: Modified band alignment for photocatalytic water splitting application by nitridation treatment. ACS Appl. Mater. Interfaces.

[18] Zhang Z., Huang B., Qian Q., Gao Z., Tang X., Li B. (2020). Strain-tunable III-nitride/Zno heterostructures for photocatalytic water-splitting: A hybrid functional calculation. APL Mater..

[19] Guo H., Zhang Z., Huang B., Wang X., Niu H., Guo Y., Li B., Zheng R., Wu H. (2020). Theoretical study of photocatalytic properties on 2D Inx(X=S, Se)/transition-metal disulfides (Mos2 and Ws2) van der waals heterostructures. Nanoscale.

[20] Deng Z., Wang X., Cui J. (2019). Effect of interfacial defects on the electronic properties of graphene/G-Gan heterostructures. RSC Adv..

[21] Liu X., Zhang Z., Luo Z., Lv B., Ding Z. (2019). Tunable electronic properties of graphene/G-AlN heterostructure: The effect of vacancy and strain engineering. Nanomaterials.

[22] Gao X., Shen Y., Ma Y., Wu S., Zhou Z. (2019). Graphene/G-Gec bilayer heterostructure: Modulated electronic properties and interface contact via external vertical strains and electric fileds. Carbon.

[23] Zhang W.X., Shi C.H., He C., Bai M. (2020). External-strain induced transition from schottky to ohmic contact in Graphene/Ins and Graphene/Janus In2SSe heterostructures. J. Solid State Chem..

[24] Padilha J.E., Fazzio A., da Silva A.J. (2015). Van der waals heterostructure of phosphorene and graphene: Tuning the schottky barrier and doping by electrostatic gating. Phys. Rev. Lett..

[25] Young A.F., Kim P. (2009). Quantum interference and klein tunnelling in graphene heterojunctions. Nat. Phys..

[26] Kwak J.Y., Hwang J., Calderon B., Alsalman H., Munoz N., Schutter B., Spencer M.G. (2014). Electrical characteristics of multilayer Mos_2_ Fet’s with Mos_2_/Graphene heterojunction contacts. Nano Lett..

[27] Duan J.H., Yang S.G., Liu H.W., Gong J.F., Huang H.B., Zhao X.N., Zhang R., Du Y.W. (2005). Preparation and characterization of straight and zigzag AlN nanowires. J. Phys. Chem. B.

[28] Liu C., Hu Z., Wu Q., Wang X., Chen Y., Sang H., Zhu J., Deng S., Xu N. (2005). Vapor−solid growth and characterization of aluminum nitride nanocones. J. Am. Chem. Soc..

[29] Nam K.B., Li J., Nakarmi M.L., Lin J.Y., Jiang H.X. (2003). Deep ultraviolet picosecond time-resolved photoluminescence studies of AlN epilayers. Appl. Phys. Lett..

[30] Tsipas P., Kassavetis S., Tsoutsou D., Xenogiannopoulou E., Golias E., Giamini S.A., Grazianetti C., Chiappe D., Molle A., Fanciulli M. (2013). Evidence for graphite-like hexagonal AlN nanosheets epitaxially grown on single crystal Ag(111). Appl. Phys. Lett..

[31] Song N., Ling H., Wang Y., Zhang L., Yang Y., Jia Y. (2019). Intriguing electronic properties of germanene/ indium selenide and antimonene/ indium selenide heterostructures. J. Solid State Chem..

[32] Sciuto A., La Magna A., Angilella G.G.N., Pucci R., Greco G., Roccaforte F., Giannazzo F., Deretzis I. (2019). Extensive fermi-level engineering for graphene through the interaction with aluminum nitrides and oxides. Phys. Status Solidi RRL.

[33] Sun M., Chou J.-P., Yu J., Tang W. (2017). Effects of structural imperfection on the electronic properties of graphene/Wse_2_ heterostructures. J. Mater. Chem. C.

[34] Li Y., Wang J., Zhou B., Wang F., Miao Y., Wei J., Zhang B., Zhang K. (2018). Tunable interlayer coupling and schottky barrier in graphene and janus mosse heterostructures by applying an external field. Phys. Chem. Chem. Phys..

[35] Kresse G., Furthmüller J. (1996). Efficient iterative schemes for ab initio total-energy calculations using a plane-wave basis set. Phys. Rev. B.

[36] Perdew J.P., Burke K., Ernzerhof M. (1996). Generalized gradient approximation made simple. Phys. Rev. Lett..

[37] Blöchl P.E. (1994). Projector augmented-wave method. Phys. Rev. B.

[38] Grimme S. (2006). Semiempirical Gga-type density functional constructed with a long-range dispersion correction. J. Comput. Chem..

[39] Zhang Z., Cao R., Wang C., Li H.-B., Dong H., Wang W.-H., Lu F., Cheng Y., Xie X., Liu H. (2015). Gan as an Interfacial passivation layer: Tuning band offset and removing fermi level pinning for III–V MOS devices. ACS Appl. Mater. Interfaces.

[40] Zhang Z., Li B., Qian Q., Tang X., Hua M., Huang B., Chen K.J. (2017). Revealing the nitridation effects on gan surface by first-principles calculation and X-Ray/ultraviolet photoemission spectroscopy. IEEE Trans. Electron Devices.

[41] Monkhorst H.J. (1976). Special points for brillonin-zone integrations. Phys. Rev. B.

[42] Liao Y., Zhang Z., Gao Z., Qian Q., Hua M. (2020). Tunable properties of novel Ga_2_O_3_ monolayer for electronics and optoelectronics applications. ACS Appl. Mater. Interfaces.

[43] Wang V., Xu N., Liu J.C., Tang G., Geng W.T. Vaspkit: A User-Friendly Interface Facilitating High-Throughput Computing and Analysis Using Vasp Code. https://arxiv.org/abs/1908.08269.

[44] Björkman T., Gulans A., Krasheninnikov A.V., Nieminen R.M. (2012). Van der waals bonding in layered compounds from advanced density-functional first-principles calculations. Phys. Rev. Lett..

[45] Zhao-Fu Z., Tie-Ge Z., Hai-Yang Z., Xiang-Lei W. (2013). First-principles calculations of 5D atoms doped hexagonal-aln sheets: Geometry, magnetic property and the influence of symmetry and symmetry-breaking on the electronic structure. Chin. Phys. B.

[46] Phuc H.V., Ilyasov V.V., Hieu N.N., Nguyen C.V. (2018). Electric-field tunable electronic properties and schottky contact of graphene/phosphorene heterostructure. Vacuum.

[47] Henkelman G., Arnaldsson A., Jónsson H. (2006). A fast and robust algorithm for bader decomposition of charge density. Comput. Mater. Sci..

[48] Khomyakov P.A., Giovannetti G., Rusu P.C., Brocks G., van den Brink J., Kelly P.J. (2009). First-principles study of the interaction and charge transfer between graphene and metals. Phys. Rev. B.

[49] Driussi F., Venica S., Gahoi A., Gambi A., Giannozzi P., Kataria S., Lemme M.C., Palestri P., Esseni D. (2019). Improved understanding of metal–graphene contacts. Microelectron. Eng..

[50] Robertson J. (2000). Band offsets of wide-band-gap oxides and implications for future electronic devices. J. Vac. Sci. Technol. B Microelectron. Nanometer Struct. Process. Meas. Phenom..

[51] Robertson J., Guo Y., Zhang Z., Li H. (2020). Extending the metal-induced gap state model of schottky barriers. J. Vac. Sci. Technol. B Microelectron..

[52] Deng S., Sumant A.V., Berry V. (2018). Strain engineering in two-dimensional nanomaterials beyond graphene. Nano Today.

[53] Si C., Lin Z., Zhou J., Sun Z. (2016). Controllable schottky barrier in gase/graphene heterostructure: The role of interface dipole. 2D Mater..

[54] Matthes L., Pulci O., Bechstedt F. (2014). Optical properties of two-dimensional honeycomb crystals graphene, silicene, germanene, and tinene from first principles. New J. Phys..

[55] Matthes L., Pulci O., Bechstedt F. (2016). Influence of out-of-plane response on optical properties of two-dimensional materials: First principles approach. Phys. Rev. B.

[56] Hüser F., Olsen T., Thygesen K.S. (2013). How dielectric screening in two-dimensional crystals affects the convergence of excited-state calculations: Monolayer Mos2. Phys. Rev. B.

[57] Liu X.-F., Luo Z.-J., Zhou X., Wei J.-M., Wang Y., Guo X., Lv B., Ding Z. (2019). Structural, mechanical, and electronic properties of 25 kinds of III–V binary monolayers: A computational study with first-principles calculation. Chin. Phys. B.

[58] Heyd J., Scuseria G.E., Ernzerhof M. (2003). Hybrid functionals based on a screened coulomb potential. J. Chem. Phys..

[59] Heyd J., Scuseria G.E., Ernzerhof M. (2006). Erratum: “Hybrid functionals based on a screened coulomb potential” [J. Chem. Phys. 118, 8207]. J. Chem. Phys..

